# The Effects of Exercise on BDNF Levels in Adolescents: A Systematic Review with Meta-Analysis

**DOI:** 10.3390/ijerph17176056

**Published:** 2020-08-20

**Authors:** Kesley Pablo Morais de Azevedo, Victor Hugo de Oliveira, Gidyenne Christine Bandeira Silva de Medeiros, Ádala Nayana de Sousa Mata, Daniel Ángel García, Daniel Guillén Martínez, José Carlos Leitão, Maria Irany Knackfuss, Grasiela Piuvezam

**Affiliations:** 1 Post-Graduate Program in Public Health, Federal University of Rio Grande do Norte, 59078-970 Natal, Brazil; victorhugoef@hotmail.com (V.H.d.O.); gidyenne@gmail.com (G.C.B.S.d.M.); adalamata@gmail.com (Á.N.d.S.M.); gpiuvezam@yahoo.com.br (G.P.); 2 Department of Sociosanitary Sciences, University of Murcia, 30100 Murcia, Spain; angelgarcia.da@gmail.com; 3 Faculty of Nursing, Catholic University of Murcia (UCAM), 30107 Murcia, Spain; dguillen@ucam.edu; 4 Center for Research in Sport, Health and Human Development, University of Trás-os-Montes and Alto Douro, 5001-801 Vila Real, Portugal; jcleitao@utad.pt; 5 Post-Graduate Program in Health and Society, State University of Rio Grande do Norte (UERN), 59610-210 Mossoró, Brazil; kmariairany@yahoo.com.br

**Keywords:** exercise, brain-derived neurotrophic factor, BDNF, adolescent, systematic review, meta-analysis

## Abstract

The aim of this study was to analyze the evidence available in the literature about the effects of exercise on brain-derived neurotrophic factor levels in adolescents. The literature searches were conducted in PubMed, Embase, Scopus, ScienceDirect, Web of Science, SportDiscus, the Cochrane Central Register of Controlled Trials (CENTRAL) and CINAHL. Randomized controlled trials and non-randomized controlled trials performed with adolescents (10–19 years) who underwent different exercise programs and who evaluated BDNF levels before and after the intervention were included. We included six studies, four RCTs and two non-RCTs in the systematic review with a total of 407 adolescents. In two randomized trials and one non-RCT, the intervention groups showed significant improvements in BDNF levels compared with the control group. The results presented in the meta-analysis indicate that despite the positive effect in favor of the intervention, there were no significant differences (standardized mean difference 0.28 ng/mL, 95% confidence interval −0.28 to 0.85; *p* = 0.32, I² = 0%). The results presented in our review indicate that aerobic exercise programs practiced in moderate- or high-intensity are promising strategies to increase BDNF levels in adolescents. However, further studies are required to support this finding.

## 1. Introduction

Adolescence is marked by biological, behavioral, and social changes that accompany brain development [1]. Regarding this, it is worth noting that some changes are enhanced during adolescence, such as prefrontal cortex maturation, neural reorganization process and cognitive development [2]. Current literature indicates that during this period brain plasticity increases and that physical exercises practice can stimulate some markers, including the brain-derived neurotrophic factor (BDNF) [3].

BDNF is understood to be an essential neurotrophin that modulates cognition, neuroplasticity, and angiogenesis and strengthens neural connectivity. Recent literature indicates that these biological activities are crucial in the development of learning and memory and contribute effectively to better academic performance and brain health [4,5].

In addition to playing an important role in several brain plasticity aspects, BDNF can act to regulate metabolic functions (fat oxidation and glucose uptake), contribute to cardiovascular improvement, and reduce the risk for neurodegenerative diseases [6]. In this sense, the available evidence in the literature indicates that obese and type 2 diabetes people may have a negative regulation of BDNF levels compared to people who exercise regularly [1,7].

In this context, experimental studies in animals and humans have shown that BDNF is a potential relationship mediator between the benefits of regular exercise and cognition. It should be noted that the results presented in several systematic reviews and meta-analyzes from experimental studies that evaluated the effects of exercise on BDNF levels indicate that acute or chronic exercise increased BDNF levels [7,8,9,10]. In turn, they emphasized that duration and intensity were positively correlated with a BDNF levels increase [11]. However, there is a gap in evidence systematization discussed in studies conducted with adolescents.

According to evidence available, a systematic review of 32 studies addressing the acute and chronic effects of exercise on young adults, adults, and the elderly found that programs with aerobic exercise promoted improvements in peripheral BDNF concentrations [6,7]. However, studies with adolescents were not included in the analysis. Strengthening the discussion around the positive effects of exercise on cognition, studies in adults have found that improvements in BDNF levels could possibly lead to hippocampal neurogenesis, therefore, enhancing executive function [7,12,13,14].

Some researchers have focused their attention on evaluating the effects of exercise programs on adolescent cognition, taking into consideration that adolescence is a phase in which the brain is still developing and the increase in BDNF may potentiate hippocampal neuronal growth and development, as well as improve cognitive capacity, learning, and memory [15,16].

It is noteworthy that recent systematic reviews have evaluated the acute and chronic effects of physical exercise on cognition in adolescents and the results pointed to positive effects in favor of the interventions [17,18,19,20]. Similarly, an umbrella review by the American College of Sports Medicine (ACSM) also found that exercise practitioners had improved cognition [21]. Regarding these studies, BDNF appears as a potential mediator of the relationship between exercise and cognition. In addition to the ACSM recommending the proposition of new studies, systematic analyses of research on the subject were not identified, as there is limited evidence in adolescents [22].

The evidence pointed out so far is supported by the findings described in the review conducted by Huang et al. and in the meta-regression systematic review by Feter et al. [6,7]. In the first review, experimental and observational studies performed in adults and elderly were analyzed and the results indicated that there were improvements in BDNF levels after the intervention period with aerobic exercises, on the other hand, habitual physical activity was inversely correlated with peripheral BDNF [7]. In turn, the study by Feter et al. discussed the dose-response relationship between BDNF concentration and the amount of physical activity or physical exercise in humans. The findings presented in this study reinforce the arguments that exercise promotes improvements in BDNF levels, and that this increase is positively correlated with its intensity [6].

Considering the promising results of the presented reviews, we can highlight that there are gaps in the literature on the possible impacts generated by exercise on BDNF levels during adolescence. It is necessary to understand how exercise acts on cognition during this period, which is marked by a peak in brain plasticity, in addition to verifying what are the potential benefits for health improvement. Based on the results described and the gaps presented in these studies, the aim of this study was to analyze the evidence available in the literature about the effects of exercise on BDNF levels in adolescents.

## 2. Materials and Methods

The systematic review and meta-analysis followed the recommendations of the Preferred Reporting Items for Systematic Reviews and Meta-Analyses (PRISMA) and the Cochrane Handbook of Systematic Reviews of Interventions [23]. The study design, containing all the planning and elaboration steps, was described in the published protocol [24]. The study was registered in the Prospective Register of Systematic Reviews (PROSPERO; CRD42018110683).

### 2.1. Eligibility Criteria

Studies that met the eligibility criteria based on Population, Interventions, Control, Outcomes, and Study design were included (PICOS) [25]; (P): studies with adolescents (10–19 years); (I): interventions with aerobic exercise programs, resistance training or concurrent training; (C): control group or other intervention; (O): studies that evaluated BDNF levels; and (S) randomized controlled trials (RCTs) and non-randomized controlled trials (non-RCTs). There was no language restriction. Studies of interventions with physical exercise programs in adolescents with psychological or neuroendocrine problems and physical or intellectual disabilities were excluded.

### 2.2. Literature Search

In the review planning stage, all possible search terms and keywords combinations were tested. We use this method to check each research strategy sensitivity and specificity applied in the databases [24]. Subsequently, the bibliographic searches were performed by two researchers (K.P.M.A. and V.H.O.) applying search strategies that combined the terms: (1) “adolescent” [Medical Subject Headings (MeSH)] OR “teenager”; (2) “exercise” [MeSH]; (3) “brain-derived neurotrophic factor” [MeSH] OR BDNF. Boolean operators AND and OR were applied. Searches were conducted in eight electronic databases (PubMed, Embase, Scopus, ScienceDirect, Web of Science, SportDiscus, the Cochrane Central Register of Controlled Trials (CENTRAL) and CINAHL) until March 3, 2020 (Supplementary Table S1). Initially, all selected articles were entered into Rayyan (mobile app for systematic reviews) and duplicates were removed [26]. The reading of titles and abstracts and, later, the full articles were carried out independently and blindly by two researchers (K.P.M.A. and V.H.O.). All differences were resolved with the help of the third researcher (G.P.). Reference lists of the inserted articles were analyzed to identify potentially eligible studies [27].

### 2.3. Data Extraction

Two reviewers (K.P.M.A. and G.C.B.S.M.) extracted data related to the authors’ name, year of publication, sample characteristics, assessed outcome (BDNF), and intervention program design (exercise type, frequency, duration, and intensity). Results of BDNF levels obtained by the intervention and control groups were extracted before and after the interventions. All discrepancies observed in this phase were resolved with the assistance of a third researcher (G.P.).

### 2.4. Assessment of Risk of Bias

To assess RCTs, version 2 of the Cochrane risk of bias tool (RoB 2.0) was applied. RoB 2.0 domains are structured to assess the risk of bias in the randomization process, deviations from intended interventions, missing outcome data, outcome measurement, selection of reported outcome, and overall bias [28]. Regarding classification, the risk of bias was assessed as low, high, or some concerns.

Non-RCTs were evaluated by the “Risk of Bias in Non-Randomized Studies—of Interventions” tool (ROBINS-I) [29]. ROBINS-I domains address risk assessment of bias before intervention (confounding and selection of participants), during intervention (classification of interventions), and after intervention (departments from intentional interventions, missing data, measurement of outcomes, selection of reported results, and overall judgment). The domains can be classified as: (1) low-risk of bias; (2) moderate-risk of bias; (3) serious-risk of bias; (4) critical-risk of bias; and (5) no information.

Two researchers (K.P.M.A. and V.H.O.) independently assessed the methodological quality of the studies and differences were resolved with the help of a third researcher (G.P.). It is noteworthy that the agreement between the evaluators was calculated using the Kappa coefficient (k = 1.00 for RCTs and k = 0.573 for non-RCTs). The Grading of Recommendations Applicability, Development and Evaluation (GRADE) guidelines were used and the evidence quality was classified as very low, low, moderate and high [30].

### 2.5. Data Synthesis and Statistics

The results obtained in assessments of BDNF levels before and after interventions were extracted to calculate the delta of variation (△) and standard deviations of variation for the intervention and control groups. Then, in the comparative analyzes the results were presented through the standardized mean differences (SMD) between the groups (intervention and control) for each included study.

To perform the meta-analysis, Review Manager 5.3 was used (The Nordic Cochrane Centre: Copenhagen, Denmark) [31], software recommended by the Cochrane Handbook for Systematic of Interventions [32]. The evaluation of heterogeneity between studies was verified by chi-square and I2 statistical tests. I2 assesses the proportion of variability between studies and levels that can be classified into low levels (0% to 25%), medium levels (above 25% to 50%), or high levels of heterogeneity (above 50%). The random effects model was used to calculate the total effect size of the studies included in the meta-analysis.

## 3. Results

### 3.1. Search Results

**Figure 1:**
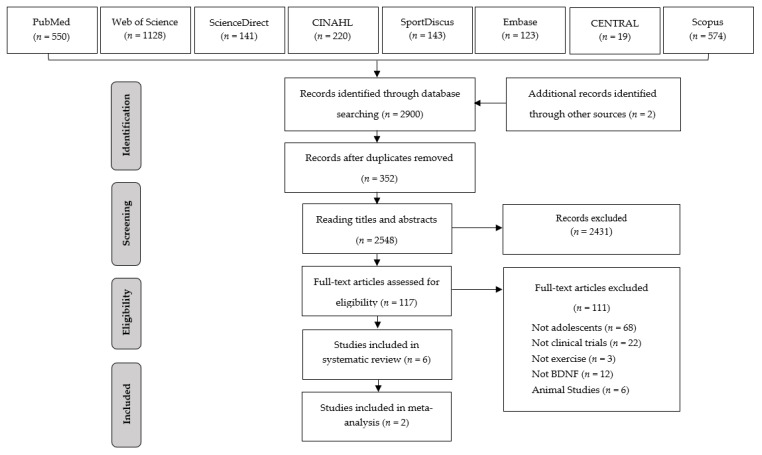
Article selection flowchart. Adapted from Preferred Reporting Items for Systematic Reviews and Meta-Analyses (PRISMA).

From the database searches 2900 articles were identified, however, 352 studies were removed because they were duplicates. Of the 2548 articles that were selected for reading titles and abstracts, only 117 were selected for reading the full texts. After applying the eligibility criteria at the stage of reading the full articles, six studies were selected for the systematic review [4,15,16,33,34,35] (Figure 1).

### 3.2. Characteristics of the Studies

The characteristics of the six studies included in this systematic review are shown in Table 1. Regarding the geographic distribution of the analyzed studies, it was observed that five were developed in South Korea [15,16,33,34,35] and one in Canada [4]. The data presented correspond to the information of the participants of the four RCTs [4,15,16,33] and two non-RCTs [34,35].

We included 409 adolescents (207 boys and 202 girls) aged 12–17 years. Regarding the Body Mass Index (BMI) and fat percentage of the participants, it was found that the studies evaluated the effects of physical exercise interventions on adolescents with normal weight [15,16,33] and overweight and obesity [4,34,35]. In four studies [4,15,16,35], adolescents were classified as irregularly active or sedentary before starting the intervention period and two studies reported no information on the level of physical activity [33,34]. In general, when addressing the types of interventions, it was observed that three studies evaluated the effect of aerobic exercise programs [15,16,34], two adopted combined exercises [33,35] and only one study compared the effects of aerobic, resistance and combined exercise programs in adolescents [4] (Table 2).

Of the six studies assessed, two RCTs [15,16] and one non-RCT [34] showed a significant improvement in BDNF levels between experimental groups and control after the intervention. The follow-up of interventions ranged from 8 weeks to 6 months, however, most studies adopted 12 weeks of training with moderate–high-intensity, a frequency of 3–5 times (per week), and duration of 20–60 min [4,15,16,33,34,35]. An important point to be highlighted is that the five studies that adopted 8–12 weeks follow up programs [15,16,33,34,35] obtained better results than the 6 month study [4].

From the results presented, it was observed that in all studies that adopted aerobic exercise programs, improvements in BDNF levels were detected, regardless of the adolescents’ nutritional status (normal weight and overweight) [4,15,16,34]. It is worth noting that greater effects were observed in groups that trained at higher intensities (moderate or high) [16]. In these studies, the control groups remained without practicing any type of exercise and followed their daily routine [4,15,34], except for the control group that stretched the entire body for 30 min during the intervention period in the study by Jeon and Ha [16].

Of the three studies that adopted 12 weeks combined exercises programs, two presented positive effects after the intervention, however, the results were not significant [33,35]. Contrary to the findings of the studies mentioned above, in the study by Goldfield et al. [4], overweight adolescents submitted to a program with combined (△ = −2.00) and resistance (△ = −1.70) exercises, a decrease in BDNF levels was found after 6 months of intervention. It is important to clarify that in these three studies [4,33,35], the adolescents allocated to the control groups did not perform any type of physical exercise.

### 3.3. Methodological Quality

In bias risk assessments, one of the RCTs [4] was rated as low-risk of bias in all domains and the other three had some concerns in the randomization process and deviations from the intended interventions [15,16,33] (Supplementary Table S2). In the assessment of non-RCTs, it was found that one of the studies was rated moderate bias [35] and the other with critical bias [34] (Supplementary Table S3). The main problems in the studies were related to confounding bias, participant selection, and intervention classification. The evidence quality from the randomized clinical trials was classified as low due to some problems in assessing the bias risk and inconsistency reasons caused by the heterogeneity degree [4,15,16,33]. In turn, serious problems were observed in the bias risk analysis and in the inconsistencies caused by heterogeneity, which reduced the classification of the non-RCTs evidence to very low [34,35].

### 3.4. Meta-Analysis of Effects on BDNF Levels

During the planning and execution stage of the meta-analysis, it was observed that the studies present different designs (RCTs and non-RCTs) and physical exercise programs (aerobic, resistance, and combined), characterizing a methodological heterogeneity. In addition, it was found that the adolescents had different physical and clinical characteristics (clinical heterogeneity). Considering the methodological and clinical heterogeneity of the selected studies, two RCTs were included in the primary analysis for evaluating the effects of aerobic exercise programs on BDNF levels in adolescents with homogeneous physical characteristics [15,16]. Regarding the type of intervention adopted in the two studies by Jeon and Ha, it should be noted that in the research carried out in 2015, the adolescents who belonged to the intervention group were submitted to an aerobic exercise program with moderate-intensity (40–60% of VO2R) and the control group did not perform any type of exercise [15].

In the study conducted in 2017, the effects of three exercise protocols practiced at different intensities were evaluated. Thus, the adolescents were allocated to a low-intensity aerobic exercise group (LIEG = 40% VO2R), moderate-intensity aerobic exercise group (MIEG = 55% VO2R) and a high-intensity aerobic exercise group (HIEG = 70% VO2R) [16]. In this study, the control group performed stretches on the entire body during the intervention period.

About the general effects of the interventions included in the meta-analysis, we found that the greatest effect (standardized mean difference (SMD) 0.63; 95% confidence interval (CI)—0.27 to 1.54) was found in the study by Jeon and Ha (2015) [15], followed by the two groups (HIEG and MIEG) from the study by Jeon and Ha (2017) [16]. On the other hand, the LIEG group obtained a lower result (SMD = −0.01; 95% CI—1.30 to 1.28) than the control group after the intervention period [16].

**Figure 2:**
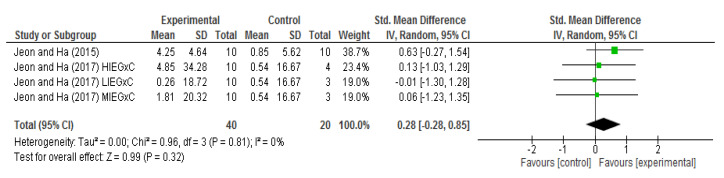
Forest plot of the BDNF levels changes (RCTs). SD = standard deviation; IV = inverse-variance; CI = confidence interval; HIEGxC = high-intensity aerobic exercise group × Control; LIEGxC = Low-intensity aerobic exercise group × Control; MIEGxC = moderate-intensity aerobic exercise group × Control.

From the comparisons before and after the intervention, it was observed that, despite the positive effect in favor of the intervention, there was no significant difference (SMD 0.28; 95% CI—0.28 to 0.85; *p* = 0.32). For this analysis, a low heterogeneity was found (I^2^ = 0%; *p* = 0.81; Figure 2).

## 4. Discussion

The results presented in our review show that adolescents undergoing aerobic exercise programs of moderate–high-intensity showed improvements in BDNF levels. In addition, the data exposed in the meta-analysis reinforce the positive effects of aerobic exercise; however, there was a small and non-significant effect size in the analyzed studies [15,16]. Overall, the evidence presented was similar to the results of a systematic review targeting young adults, adults, and the elderly [7].

It has been reported in recent systematic reviews that BDNF is a potential mediator for explaining the effects of exercise on adolescent cognition. From this perspective, the evidence available in this review indicates that aerobic exercise promoted improvements in BDNF levels in normal weight [15,16,33] and obese adolescents [4,34,35]. This fact has attracted our attention because the increase in BDNF levels enhances hippocampal neuronal growth and development, increasing cognitive ability, learning ability, and memory. These improvements positively impact educational performance and contribute to healthy brain development during adolescence.

Studies that tested aerobic exercise in obese adolescents showed better results in BDNF levels after interventions [4,15,16]. Regarding these studies, it is worth mentioning that only in the study by Jeon and Ha (2017), the control group performed daily stretching activities in different parts of the body [16]. This fact may have caused changes in BDNF levels in the post-intervention assessment. Regarding these findings, it is noteworthy that the action of BDNF bypasses the benefits related to the brain; it is involved in of metabolic functions regulation, such as fat oxidation and glucose uptake, thus, it is understood that it can be negatively regulated in those with obesity and type 2 diabetes [4,36,37].

In respect to the effects of the interventions that adopted combined exercise programs [33,35], the results were contradictory, as there were improvements in two groups and one showed lower results than the control group after the intervention period [4]. On the other hand, when we observed all the studies included in our review, only one group of adolescents underwent resistance training and, after a period of 6 months, BDNF levels were lower than the control group [4].

In a meta-analysis of 25 studies (*n* = 2152) assessing the effects of exercise on healthy and unhealthy adults, improvements in BDNF levels were observed after the intervention period. It is worth noting that this increase occurred regardless of age and health status [6].

Similarly, a systematic review assessed the effect of acute or chronic physical exercise on peripheral BDNF levels in healthy elderly people with different pathologies. Of the six studies included in the review, five showed positive results and three showed significant improvements in BDNF levels (*p* < 0.05) [38]. These findings corroborate the evidence available in the literature that exercise may be beneficial for human cognition, regardless of age.

Given this evidence, knowledge gaps related to the effects of exercise on BDNF levels as a potential mediator in cognitive improvements in adolescents are now being addressed, having been discussed in two systematic reviews and three meta-analyses [17,18,19,20,39]. Corroborating this evidence, the results presented in our study highlight the positive effects of aerobic exercise programs with moderate- or high-intensity on BDNF levels in adolescents [15,16].

When dealing with intensity issues, recent studies have used blood lactate to monitor exercise intensity. Similar to our findings, these studies indicate that intensity and lactate concentrations increase is associated with plasma and serum BDNF levels improvements. Despite the limitations described in the literature on the interaction between lactate and BDNF levels, the mechanisms may be related to increased lactate-regulated NMDA receptor activation and, as a consequence, increased intracellular calcium levels, a signaling cascade initiated by lactate binding to different G protein coupled receptors (GPCR) and through the activation of the silent information regulator 1 (SIRT1) of the PGC1α/FNDC5/BDNF pathway [11].

About the effects of different exercise programs, there was a major discussion in the literature about studies that indicate the possible dose-response mechanisms in relation to the effects promoted on cognition. Above all, it should be noted that most studies direct their attention to modifiable factors (sex and genotypes) and, as a consequence, few address issues related to prescription. This is a point to be better addressed in the studies so that we can understand the factors that can cause inter-individual heterogeneity in exercise response. There are indications in the literature suggesting that additional studies should adopt an adapted exercise prescription with comparable dosage among participants [40].

Regarding the inter-individual variability observed in the results of the studies, it is necessary to understand that when inserting “responder” and “non-responder” participants, it can interfere in the physical exercise impact on physiological parameters and brain. Therefore, there are recommendations that guide quality (type) and quantity (intensity, duration, and volume) adaptations of the exercises program, with the objective of enhancing the neuroplastic and preventive effects. In this context, we understand that high-intensity interval training (HIIT) can be considered a safe and effective strategy, in addition the insertion of higher intensities and lower volumes, can reach a greater number of responders, including the elderly and patients with chronic diseases [41].

In this context, a randomized clinical trial demonstrated that a group of elderly people who underwent a dance intervention obtained superior results in BDNF levels when compared to the group that practiced sports. These improvements have been accompanied by a gray matter volume increase [42]. A recent review conducted by Stillman et al. discussed the possible mechanisms that explain the impact of exercise on cognition. According to the review, physical exercise promotes positive effects on the hippocampus volume and improves cognitive functions related to it [22].

Regarding the practical recommendations of the findings systematized in this review, it was noted that there was a heterogeneity in the time, frequency and duration of interventions in the protocols of physical exercise applied. However, based on the evidence presented and the protocols that showed significant results at the end of the interventions, the programs composed of moderate–high-intensity aerobic exercises, a frequency of 3–5 times a week and duration of 20–60 min were the most effective strategies for increasing BDNF levels, and consequently, for better adolescent brain development and health. The recommendations presented are in line with the ACSM guidelines [21].

### 4.1. Practical Applications

Based on the evidence presented in our review, we can suggest that future research should focus on randomized clinical trials and the standardization of exercise programs with higher intensities (moderate–vigorous). In addition, we indicate that researchers can explore the possible impacts of BDNF on cognition and health in adolescents.

Another point to be considered is the knowledge gap about the impacts that obesity and other chronic diseases (e.g., type 2 diabetes) can cause on BDNF levels and consequently on cognition. For all these reasons, the need for further laboratory studies and in other contexts, such as schools and sports initiation programs, becomes evident. These results may assist in interventions planning and applicability aimed brain full development during adolescence, as well as for the maintenance of a healthy lifestyle.

### 4.2. Limitations

The main limitations of our review were related to the number of studies included, the methodological heterogeneity of the studies (RCTs and non-RCTs) and the exercise programs (aerobic, resistance and combined), the clinical heterogeneity of the adolescents (normal weight, overweight and obesity), and sample sizes. All of these factors hindered some of the intended statistical analyzes and for that reason, only two RCTs with similar characteristics were included in the meta-analysis [15,16].

In respect to the number of studies included in our study, we cannot afford the possibility that some studies may not have been captured by the search strategy adopted [24]. However, we emphasize that the methods applied in our review potentially reduce the chances of an eligible study not being included. Regarding the methodological quality of the included studies, some sources of problematic bias were observed in non-RCTs [34,35]. Meanwhile, the randomized controlled trials were classified as low-risk of bias in most domains and some concerns in the domain of the randomization process [4,15,16,33].

In addition to these aspects, it has become evident that the effects of exercise on BDNF levels in humans can be affected by the blood collection method, age, sex, health status, the use of tobacco, and alcohol consumption [6,43]. These factors should be taken into account when planning future studies on the theme.

Another important point to be highlighted, and that can cause limitations in the extrapolation of the results presented in this review, is that most studies were developed in South Korea [15,16,33,34,35]. This fact can limit the generalization of results, since cultural and social factors can alter results specifically within the scope of certain interventions or treatments [44].

## 5. Conclusions

The evidence available in this review allows us to conclude that aerobic exercise programs practiced with moderate–high-intensity (3–5 times a week and lasting 20–60 min) are promising strategies to increase BDNF levels in adolescents. In addition, these programs have also shown positive results for overweight and obese adolescents. However, additional studies are needed to strengthen the evidence.

## Figures and Tables

**Table 1:** Characteristics of the studies included.

Author (Year)	Country	Design	Participants	BMI	Training Status
Goldfield et al. (2018) [4]	Canada	RCT (Parallel-group)	282 (84 boys, 198 girls) Aerobic: *n* = 69 (15.5 ± 1.3 y) Resistance: *n* = 70 (15.8 ± 1.5 y) Combined: *n* = 74 (15.5 ± 1.3 y) Control: *n* = 69 (15.6 ± 1.3 y)	Aerobic = 34.6 ± 4.2 Resistance = 35.3 ± 4.8 Combined = 34.5 ± 4.1 Control = 34.3 ± 5.0	Irregularly active
Jeon and Ha (2015) [15]	South Korea	RCT (Parallel-group)	20 (boys) Exercise: *n* = 10 (15 y) Control: *n* = 10 (15 y)	Exercise = 19.8 ± 4.6 Control = 18.9 ± 3.8	Irregularly active
Jeon and Ha (2017) [16]	South Korea	RCT (Parallel-group)	40 (boys) Low-intensity: *n* = 10 (15.06 ± 0.7 y) Moderate-intensity: *n* = 10 (15.47 ± 0.8 y) High-intensity: *n* = 10 (15.15 ± 0.3 y) SG: *n* = 10 (15.05 ± 0.4 y)	LIEG = 18.05 ± 1.88 MIEG = 17.15 ± 2.01 HIEG = 18.34 ± 2.54 SG = 19.35 ± 1.44	Sedentary
Kim et al. (2015) [33]	South Korea	RCT (Parallel-group)	30 boys Combined group: *n* = 15 (10.93 ± 0.3 y) Control group: *n* = 15 (11.00 ± 0.0 y)	Combined group = 17.90 ± 2.09 Control group = 18.15 ± 1.47	ND
Lee et al. (2014) [34]	South Korea	Non-RCT (Parallel-group)	19 (15 boys, 4 girls) Obesity group: *n* = 8 (16.3 ± 0.9 y) Control group: *n* = 11 (16.4 ± 1.4 y)	Obesity group = 27.47 ± 2.51 Control group = 22.35 ± 3.94	ND
Shim and Kim (2012) [35]	South Korea	Non-RCT (Parallel-group)	18 boys Exercise group: *n*= 9 (13.0 ± 0.71 y) Control group: *n*= 9 (12.78 ± 0.83 y)	Exercise group: 27.0 ± 2.39 Control group: 27.0 ± 2.88	Sedentary

BMI = Body Mass Index; ND = Not Described; RCT = randomized controlled trials; Non-RCT = non-randomized controlled trials; y = years; SG = stretching group.

**Table 2:** Characteristics of the RCTs and non-RCTs included in the review.

Author (Year)	Follow-Up	Intervention	Control	Outcomes	Main Results
Goldfield et al. (2018) RCT [4]	6 months (4 days per week)	(1) Aerobic (20–45 min, 65–85% HR_max_) (2) Resistance (20–45 min, exercises with 2 × 15 progressing to 3 × 8, 8RM) (3) Combined (Complete aerobic training program plus resistance training program during each session)	The control group received only dietary counselling with no exercise prescription	Serum BDNF (ng/mL)	No significant within- or between-group changes in BDNF Aerobic group (△ = +1.80) Resistance group (△ = −2.00) Combined group (△ = −1.70)
Jeon and Ha 2015) RCT [15]	8 weeks (3 days per week)	Exercise was performed on treadmills and the exercise intensity was set between 40% and 60%VO2R	The control group were asked to continue their daily normal and sedentary activities	Serum BDNF (pg/mL)	The exercise group showed a significant increase in BDNF levels (*p* < 0.001) Exercise (△ = +4.25) Control (△ = +0.85)
Jeon and Ha (2017) RCT [16]	12 weeks (4 days per week)	(1) Low-intensity aerobic exercise (43.34 ± 3.59 min, 40% VO2R) (2) Moderate-intensity aerobic exercise (33.33 ± 3.64 min, 55% VO2R) (3) High-intensity aerobic exercise (25.76 ± 2.10 min, 70% VO2R)	The SG performed whole-body stretching for 30 min at the same time, frequency and location as the aerobic exercise groups.	Serum BDNF (ng/mL)	The MIEG (*p* < 0.05) and HIEG (*p* < 0.01) groups showed a significant increase in BDNF levels LIEG (△ = +0.26) MIEG (△ = +1.81) HIEG (△ = +4.85) SG (△ = +0.54)
Kim et al. (2015) RCT [33]	12 weeks (5 days per week)	Combined exercise group (CEG): taekwondo movement-based exercise program (60 min, 50–80% HR)	No exercise	Serum BDNF (ng/mL)	The CEG showed improvements in BDNF levels, but there was no significant difference between the groups Combined group (△ = +2.58) Control group (△ = +0.07)
Lee et al. (2014) Non-RCT [34]	12 weeks (3 days per week)	(1) Obesity group: aerobic exercise (40–60 min, 40–60% VO_2max_)	No exercise	Serum BDNF (pg/mL)	Only GO showed a significant increase in BDNF levels (*p* < 0.05) Obesity group (△ = +11.62) Control group (△ = −0.51)
Shim and Kim (2012) Non-RCT [35]	12 weeks (3 days per week)	(1) Exercise group (20–25 min, 55–75% HR_max_) and resistance (20–45 min, exercises with 3 × 8 progressing to 3 × 15, 50–70% 1RM)	No exercise	Serum BDNF (ng/mL)	The EG showed improvements in BDNF levels, but there was no significant difference between the groups Exercise group (△ = +2.58) Control group (△ = +0.07)

BDNF= Brain-Derived Neurotrophic Factor; HR = heart rate; HR_max_ = maximum heart rate; VO_2max_ = maximum oxygen consumption; VO2R = reserve oxygen consumption; RM = maximum resistance; LIEG = Low-intensity aerobic exercise group; MIEG = moderate-intensity aerobic exercise group; HIEG = high-intensity aerobic exercise group; SG = Stretching group.

## Supplementary Materials

The following are available online at https://www.mdpi.com/1660-4601/17/17/6056/s1, Supplementary Table S1: Search strategies for each database, Supplementary Table S2: Risk of bias in randomized controlled trials (Rob 2.0), Supplementary Table S3: Risk of bias in non-randomized controlled trials (ROBINS-I).

## Abbreviations

ACSM	American College of Sports Medicine
BDNF	Brain-derived neurotrophic factor
BMI	Body mass index
HIEG	High-intensity aerobic exercise group
HR	Heart rate
HRmax	Maximum heart rate
LIEG	Low-intensity aerobic exercise group
MIEG	Moderate-intensity aerobic exercise group
Non-RCTs	Non-randomized controlled trials
PRISMA	Preferred Reporting Items for Systematic Reviews and Meta-Analyses
RCTs	Randomized controlled trials
RM	Maximum resistance
RoB	Risk of bias
ROBINS-I	Risk of Bias in Non-Randomized Studies—of Interventions
SMD	Standardised mean differences
VO2max	Maximum oxygen consumption
VO2R	Reserve oxygen consumption
