# Supplementary material for: The Effects of Exercise on BDNF Levels in Adolescents: A Systematic Review with Meta-Analysis
Source: Int J Environ Res Public Health. 2020 Aug 20;17(17):6056. doi: 10.3390/ijerph17176056 (PMC7503913; doi:10.3390/ijerph17176056)
Supplement: Supplementary file 1 [file ijerph-17-06056-s001.pdf]

Review

The effects of exercise on BDNF levels in adolescents: A Systematic Review with Meta-Analysis

Kesley Pablo Morais de Azevedo [1], Victor Hugo de Oliveira [1], Gidyenne Christine Bandeira Silva de Menezes [1], Ádala Nayana de Sousa Mata [1], Daniel Ángel García [2], Daniel Guillén Martínez [3], José Carlos Leitão [4], Maria Irany Knackfuss [5] and Grasiela Piuvezam [1]

Table S1. Search strategies for each database.

Database	Search Strategies	Number of Articles
SCOPUS	("exercise") AND ("BDNF" OR "Brain Derived Neurotrophic Factor") AND ("adolescent" OR "teenager") AND (LIMIT-TO (DOCTYPE, "ar") OR LIMIT-TO (DOCTYPE, "sh"))	(*n* = 574)
PUBMED	("exercise") AND ("BDNF" OR "Brain Derived Neurotrophic Factor") AND ("adolescent" OR "teenager")	(*n* = 550)
WEB OF SCIENCE	TS= ("exercise" AND "BDNF" OR "Brain Derived Neurotrophic Factor" AND "adolescent" OR "teenager" AND "clinical trials") NOT TS=animals<br>Refinado por: TIPOS DE DOCUMENTO: (ARTICLE OR EARLY ACCESS)<br>Índices=SCI-EXPANDED, SSCI, A&HCI, CPCI-S, CPCI-SSH, ESCI Tempo estipulado= Todos os anos	(*n* = 1128)
SCIENCEDIRECT	("exercise") AND ("BDNF" OR "Brain Derived Neurotrophic Factor") AND ("adolescent" OR "teenager")<br>Refinado por: TIPOS DE DOCUMENTO: (ARTICLE OR EARLY ACCESS)	(*n* = 141)
COCHRANE	("exercise") AND ("BDNF" OR "Brain Derived Neurotrophic Factor") AND ("adolescent OR teenager")	(*n* =19)
EMBASE	(exercise) AND (BDNF OR Brain Derived Neurotrophic Factor) AND (adolescent OR teenager)	(*n* = 123)
SPORTDiscus	("exercise" AND "BDNF" OR "Brain Derived Neurotrophic Factor" AND "adolescent" OR "teenager" AND "clinical trials")	(*n* = 143)
CINAHL	("exercise" AND "BDNF" OR "Brain Derived Neurotrophic Factor" AND "adolescent" OR "teenager" AND "clinical trials")	(*n* = 220)

Table S2. Risk of bias in RCTs (Rob 2.0).

	Randomization Process	Deviations from the Intended Interventions	Missing Outcome Data	Measurement of the Outcome	Selection of the Reported Result	Overall Bias
Goldfield et al. (2018) [4]	Low	Low	Low	Low	Low	Low
Jeon and Ha (2017) [15]	Some concerns	Low	Low	Low	Low	Some concerns
Jeon and Ha (2015) [16]	Some concerns	Low	Low	Low	Low	Some concerns
Kim et al. (2015) [33]	Some concerns	Some concerns	Low	Low	Low	Some concerns

Table S3. Risk of bias in non-RCTs (ROBINS-I).

	Confounding	Selection of Participants	Classification of Interventions	Departures from Intended Interventions	Missing Data	Measurement of Outcomes	Selection of Reported Results	Overall Judgment
Lee et al. (2014) [34]	Serious	Low	Low	Low	Critical	Low	Low	Critical
Shim and Kim (2012) [35]	Low	Low	Moderate	Low	Low	Low	Low	Moderate
